# Reining in the CD8+ T cell: Respiratory virus infection and PD-1-mediated T-cell impairment

**DOI:** 10.1371/journal.ppat.1007387

**Published:** 2019-01-03

**Authors:** Meredith C. Rogers, John V. Williams

**Affiliations:** 1 Department of Pediatrics, University of Pittsburgh School of Medicine, Pittsburgh, Pennsylvania, United States of America; 2 Department of Pathology, Microbiology, and Immunology, Vanderbilt University Medical Center, Nashville, Tennessee, United States of America; 3 UPMC Children’s Hospital of Pittsburgh, Pittsburgh, Pennsylvania, United States of America; University of Kentucky, UNITED STATES

## Introduction

Viral acute respiratory infections (ARIs) are associated with cluster of differentiation (CD)8^+^ T cells that exhibit diminished production of cytokines and cytotoxic molecules. Though these cells recognize major histocompatibility complex (MHC)-I–restricted viral epitopes, ex vivo stimulation of these cells with these same viral peptides fails to elicit production of interferon (IFN)γ, interleukin (IL)-2, or tumor necrosis factor (TNF)-α; degranulation as measured by CD107a staining; or other features of functional CD8^+^ T cells. This is a unique feature of viral ARI because T-cell dysfunction occurs in chronic rather than acute infections of many other organs. Although CD4^+^ regulatory T cells (Tregs) contribute to CD8^+^ T-cell dysfunction, recent work on a variety of viruses has identified inhibitory receptors as a key mediator of this phenotype. Programmed cell death 1 (PD-1) is the most well-studied inhibitory receptor in T-cell impairment, but there is growing evidence that other inhibitory receptors also play a role. The tendency of CD8^+^ T cells to have significantly reduced functionality in the context of respiratory virus infection has been termed "T cell impairment".

## What is T-cell impairment?

**Fig 1:**
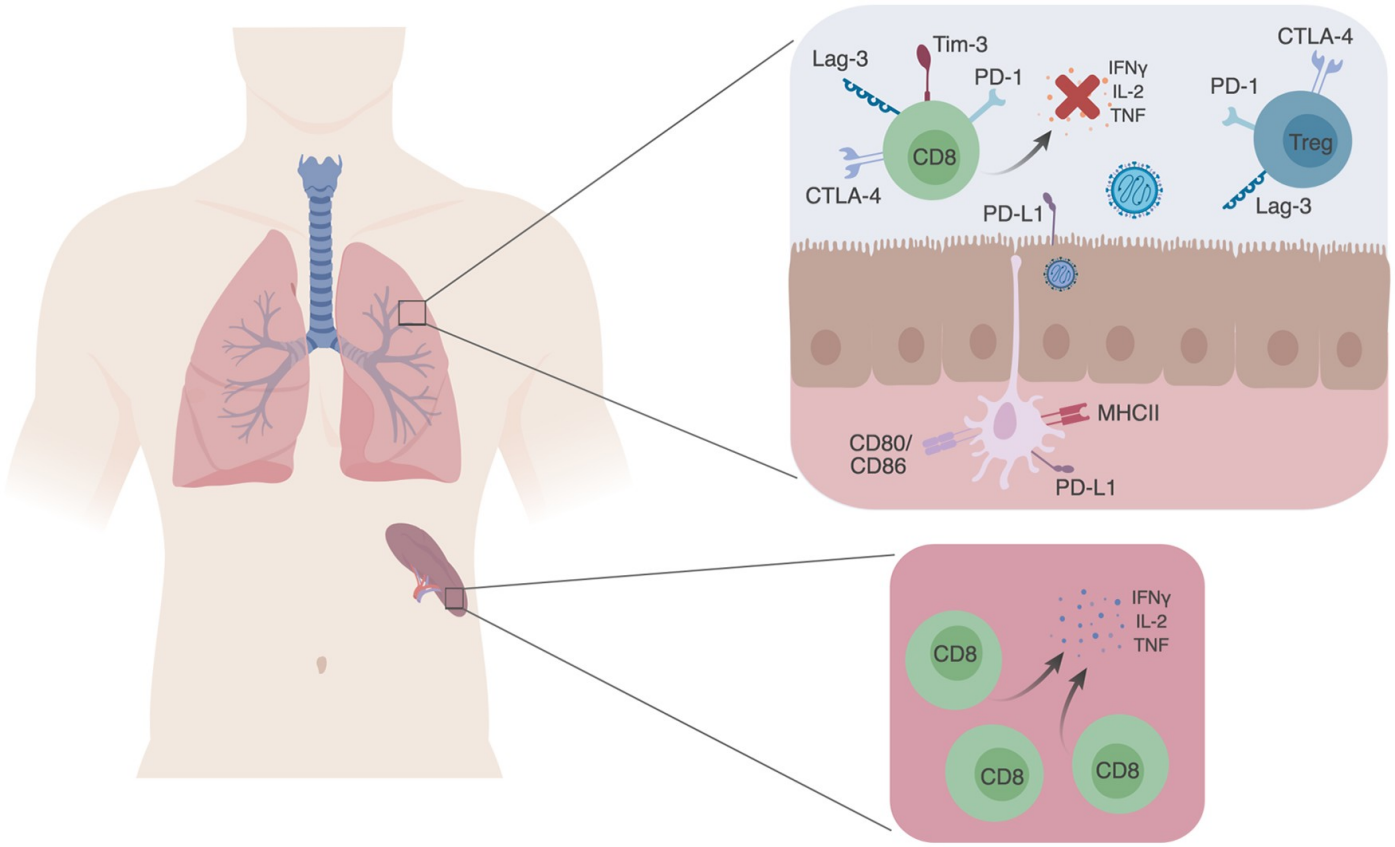
Respiratory virus infection is associated with T-cell impairment. During respiratory virus infection, impaired virus-specific T cells in the lung fail to produce pro-inflammatory cytokines or perform cytotoxic functions. T-cell impairment is at least partially mediated by expression of inhibitory receptors, which decrease effector T-cell function and promote Treg activity. In contrast, virus-specific T cells in the spleen rarely express inhibitory receptors and are functional. PD-1 and other inhibitory receptors are expressed in the lung during both primary infection and reinfection. CD8, cluster of differentiation 8; CTLA-4, cytotoxic T-lymphocyte-associated protein 4; IFN_Ɣ_, interferon gamma; IL-2, interleukin 2; Lag-3, Lymphocyte-activation gene 3; MHCII, major histocompatibility complex class II; PD-1, programmed cell death 1; PD-L1,; Tim-3, T-cell immunoglobulin and mucin-domain containing-3; TNF, tumor necrosis factor; Treg, regulatory T cells.

T-cell immunity, especially CD8^+^ T cells, is essential to clearing acute viral lung infections. In mouse models, absence of CD8^+^ T cells leads to delayed clearance of viruses, whereas humans that have defects in T-cell immunity associated with aging, immune suppression, or cancer tend to have more severe infections and poorer outcomes [1, 2]. However, despite this clear need for CD8^+^ T-cell–mediated immunity, infections due to a broad range of acute viruses, including influenza virus, respiratory syncytial virus (RSV), pneumonia virus of mice, vaccinia virus (respiratory but not systemic infection), human metapneumovirus (HMPV), and others, have been associated with a state called T-cell impairment [3–11]. Broadly defined, T-cell impairment occurs when virus-specific CD8^+^ T cells fail to produce inflammatory cytokines or perform cytotoxic functions at the site of infection, while the same virus-specific cells at other sites, such as the spleen, are completely functional (Fig 1). Epitope-specific cells in the lung-draining mediastinal lymph nodes are completely functional [7]. This suggests that the inflammatory environment of the lung parenchyma drives the process of T-cell impairment during ARI.

T-cell impairment requires cognate antigen, because CD8^+^ T cells that are not specific to a viral antigen remain functional during primary infection [4]. It also appears to require active infection, because dendritic cell vaccination leads to functional virus-specific CD8^+^ T cells [4]. However, adoptive transfer of cells into a naïve lung causes the transferred cells to lose some effector potential, though infection further decreases the function of these cells, indicating that the lung environment itself may program some degree of suppression even in settings without infection [12, 13]. Impairment also occurs during secondary infections [3–7, 14], which may indicate one reason why humans have poor T-cell memory to respiratory viruses and are susceptible to reinfection.

## Why would the lung favor T-cell impairment?

As the site of gas exchange, the lung is essential for the long-term survival of an organism. Left unchecked, effector T cells, inflammatory cytokines, and other immune mediators could damage healthy tissue alongside virus-infected cells. Indeed, a significant portion of lung injury after serious infections is due to immunopathology rather than to the virus infecting and killing cells [15, 16]. Therefore, it is necessary for the immune system to balance virus clearance and immune-mediated damage during infection, and CD8+ T-cell impairment likely represents a host mechanism of protective immunoregulation.

Similarly, once a respiratory virus is cleared, normal lung homeostasis must be restored. T-cell impairment likely represents a regulatory mechanism to restore the normal state by reducing function or survival of cytotoxic virus-specific CD8^+^ T cells. Some strategies to reverse T-cell impairment, discussed below, can be associated with increased immunopathology or disease severity and delayed recovery from weight loss [4, 17, 18] at time points after viral inoculation when the virus has almost entirely been cleared.

Another tissue that appears to favor T-cell impairment is the brain. Some neurotropic infections, such as the JHM strain of murine coronavirus, cause dysfunctional virus-specific T cells [19]. Considering the critical and delicate nature of neural tissue and the limited healing capacity of the brain, it seems likely that T-cell impairment in both organs represents an adaptation to preserve vital organs during infection and recovery. Reversal of T-cell dysfunction in neurotropic infection also causes increased immunopathology and disease severity [19].

## What mediates T-cell impairment?

Much work remains to fully elucidate the mechanisms of impairment. One essential component is the action of inhibitory receptors, the most well-described being PD-1. PD-1 and other inhibitory receptors interfere with signaling through the T-cell receptor and thus have far reaching consequences on T-cell function, from cytokine release to metabolism [20–22]. PD-1 signaling alters metabolism in multiple ways: it suppresses PI3k (phosphatidylinositide 3-kinase/Akt and mTOR (mammalian target of rapamycin) activation, diverts metabolic pathways away from glycolysis and towards fatty acid oxidation, and increases reactive oxygen species that promote apoptosis [21, 23, 24]. PD-1 is expressed on T and B cells, as well as occasionally on antigen-presenting cells (APCs) [25]. PD-1 has two ligands, PD-L1 (expressed on almost all cell types) and PD-L2 (expressed on APCs and B cells). PD-1 is up-regulated by antigen stimulation, while inhibitory ligands are induced by interferons [25]. Because antigen stimulation and interferon release tend to occur simultaneously only at the site of infection, this facilitates impairment specifically in the infected tissue and explains why T cells in the spleen or lymph node are not impaired in ARI.

Blockade of PD-L1 or genetic depletion of PD-1 increases the functionality of virus-specific CD8^+^ T cells. In infections such as HMPV or influenza, blockade also leads to faster virus clearance [4, 26]. Other inhibitory receptors, including Lag-3, Tim-3, 2B4, and CTLA-4 are up-regulated in response to respiratory virus infection and appear to contribute to some degree of T-cell impairment, but thus far PD-1 appears to be the dominant inhibitory receptor for ARI [14]. Lag-3 mediates later T-cell impairment, but blockade of Lag-3 increases immunopathology without reducing viral titer [17]. Although other inhibitory receptors have been discovered and characterized, to date, they have not been evaluated for a role in T-cell impairment in viral ARI.

## Is T-cell impairment the same as T-cell exhaustion?

Although many of the same inhibitory receptors have been implicated in both impairment and exhaustion, these two states differ in a few significant ways. T-cell exhaustion is defined by antigen-unresponsive T cells after a prolonged antigenic stimulus, such as during chronic infections or cancer. Inhibitory receptor blockade and other strategies to restore T-cell function are licensed strategies for cancer treatment and are in clinical trials for chronic infection. T-cell exhaustion has been well characterized using the lymphocytic choriomeningitis virus (LCMV) model of chronic infection and is associated with progressive hierarchical loss of T-cell function [27] as well as virus persistence. Pulmonary T-cell impairment is also associated with dysfunction of multiple T-cell capacities (IL-2, TNF, granzyme B, and IFNγ production, and degranulation as measured by CD107a,) and defects in virus clearance [4].

However, one major difference between T-cell impairment and exhaustion is timing. T-cell exhaustion requires long-term antigen exposure before inhibitory receptors become up-regulated. Acute LCMV infection is associated with transient PD-1 expression, whereas chronic LCMV infection leads to sustained PD-1 expression on epitope-specific T cells [28]. In contrast, T-cell impairment is seen relatively early (day 7–8) in the adaptive immune response to ARI, and PD-1 expression remains high on lung lymphocytes weeks after virus is cleared [4]. Additionally, during T-cell exhaustion, there are subsets of exhausted T cells that can be rescued by anti–PD-1 blockade or that remain terminally differentiated and unresponsive to PD-1 blockade [29, 30]. It is not yet known whether similar subsets of impaired T cells exist in the lung.

It seems likely that the particular inflammatory environment of the pulmonary tissue triggers the cascade of PD-1 and PD-L1/2 up-regulation more rapidly, accounting for a level of impairment at day 7 of acute infection that by microarray bears a striking similarity to exhaustion at day 30 of chronic LCMV [14, 28]. For instance, alveolar macrophages express high levels of PD-L1 even at baseline [31], which may allow the lung to rapidly trigger impairment, whereas induction of PD-L1 on other APCs requires infection and inflammation.

## How might our knowledge of T-cell impairment impact drug or vaccine design?

It is important to consider lung T-cell impairment when developing vaccines or therapeutics for respiratory viruses as well as anticipating potential side effects of PD-L1 antibodies and other checkpoint blockade therapies for cancer.

There are no licensed vaccines against many common respiratory viruses (including RSV, HMPV, and parainfluenza viruses) despite extensive research. Vaccines that elicit T-cell immunity are thought to enhance protection against respiratory viruses, but whether T-cell impairment contributes to the failure of successful vaccine development in humans requires further study. The vaccine adjuvant alum induced high PD-1 expression on CD8^+^ T cells in a mouse model of influenza [32], and virus-like particle vaccination of mice against HMPV did not protect from CD8^+^ T-cell impairment and inhibitory receptor expression in secondary challenge [33]. T-cell impairment may represent a barrier to vaccination for ARI, but pharmacologic restoration of T-cell function can have detrimental effects in the lungs as well.

A rare but serious and often fatal side effect of checkpoint inhibitors is pneumonitis, or inflammation of the lung [34]. Although an exact mechanism has not been established, aberrant T-cell activation to self or foreign antigen in the lung may contribute. Humans receiving checkpoint inhibition therapy who then acquire common respiratory viral infections may be at increased risk for pneumonitis. A future improvement to checkpoint therapy would be the ability to specifically target tumor-specific T cells. Moreover, studies of respiratory virus infection in patients treated with checkpoint inhibitors would help us understand and anticipate potential risks associated with these treatments.

## Conclusion

T-cell impairment, mediated by PD-1 and other inhibitory receptors, represents a regulatory adaptation by the immune system to preserve healthy lung tissue during acute viral infection. Though antibody blockade of PD-1 and other strategies to reverse T-cell impairment offer exciting treatments for cancer, the use of these drugs can pose risks to lung function and therefore warrant further study. Pulmonary T-cell impairment contributes to memory and vaccine responses, though the ramifications of these interactions are not yet clear. Finding the perfect balance between immunoprotection and immunopathology remains elusive.
